# Methods for Improving Human Gut Microbiome Data by Reducing Variability through Sample Processing and Storage of Stool

**DOI:** 10.1371/journal.pone.0134802

**Published:** 2015-08-07

**Authors:** Monika A. Gorzelak, Sandeep K. Gill, Nishat Tasnim, Zahra Ahmadi-Vand, Michael Jay, Deanna L. Gibson

**Affiliations:** Department of Biology, University of British Columbia Okanagan, Kelowna, BC, V1V 1V7, Canada; Charité, Campus Benjamin Franklin, GERMANY

## Abstract

Gut microbiome community analysis is used to understand many diseases like inflammatory bowel disease, obesity, and diabetes. Sampling methods are an important consideration for human microbiome research, yet are not emphasized in many studies. In this study, we demonstrate that the preparation, handling, and storage of human faeces are critical processes that alter the outcomes of downstream DNA-based bacterial community analyses via qPCR. We found that stool subsampling resulted in large variability of gut microbiome data due to different microenvironments harbouring various taxa within an individual stool. However, we reduced intra-sample variability by homogenizing the entire stool sample in liquid nitrogen and subsampling from the resulting crushed powder prior to DNA extraction. We experimentally determined that the bacterial taxa varied with room temperature storage beyond 15 minutes and beyond three days storage in a domestic frost-free freezer. While freeze thawing only had an effect on bacterial taxa abundance beyond four cycles, the use of samples stored in RNAlater should be avoided as overall DNA yields were reduced as well as the detection of bacterial taxa. Overall we provide solutions for processing and storing human stool samples that reduce variability of microbiome data. We recommend that stool is frozen within 15 minutes of being defecated, stored in a domestic frost-free freezer for less than three days, and homogenized prior to DNA extraction. Adoption of these simple protocols will have a significant and positive impact on future human microbiome research.

## Introduction

Gut microbiome dynamics are key to understanding many modern non-communicable diseases such as ulcerative colitis, obesity, metabolic syndrome, and diabetes (reviewed in Chan *et al* [1]). The faecal matrix is a complex environment comprising of inorganic and organic components where the bacterial biomass is the major component of the later fraction [2]. Microbial DNA extracted from faeces is used to investigate the intestinal microbiome and has been demonstrated to be a useful proxy of distal colon microbiome [3,4]. As a result, studies examining human microbial community structure and function derived from stool samples have become prevalent. Considering that DNA can be degraded through oxidation, hydrolysis and enzymatic degradation [5], sampling methods are arguably the most important step in human microbiome studies. Yet, stool collection and storage methods are often addressed sparingly in methods sections. Collection procedures and storage conditions should be optimized to slow DNA degradation in samples and standardized to reduce the large variability seen in microbiome analyses facilitating comparisons of results from various studies.

While relatively understudied, variability in microbial community structures as a result of different sample preparation methods of human faeces has been considered. Wu *et al* [6], identified differences in bacterial taxa between replicate samples subsampled 1 cm apart on a single stool. In line with this observation, others have reported large variability of microbes within stool detected using next generation sequencing [4,7]. It is unclear if room temperature has an effect on bacterial DNA-based detection from faecal samples considering one study reports a 10% change in bacterial community [8], and another study reports no effect on phylogenetic diversity [9]. While a study has shown that stool storage at -20°C for 53 days results in the major phyla of bacteria found in stool, Firmicutes and Bacteroidetes, being altered [10], the effect of storage in a domestic frost-free freezer is less clear. Considering that faecal samples are typically stored by study participants in their domestic freezers, most often with frost free cycling, prior to being transported to the lab, it is particularly important to examine the storage effects of stool in domestic frost-free freezers.

Here we sought to determine recommendations for optimal processing of human stool for bacterial detection using DNA-based techniques like quantitative (q)PCR. We tested the homogeneity of bacterial taxa in a stool by examining five subsamples of a single stool from four different individuals. We discovered that large variation resulted from subsampling in all individual stool samples and that the inner and outer microenvironments of a stool harboured different abundances of various bacteria. To capture the community of bacteria in its entirety from one individual stool, we homogenized the stool in liquid nitrogen into a fine powder and found that subsequent subsampling resulted in significantly reduced variability. We experimentally determined that the bacterial taxa varied with room temperature storage beyond 15 minutes and beyond three days storage in a domestic frost-free freezer. While freeze thawing only had an effect on bacterial taxa abundance beyond four cycles, the use of samples stored in RNAlater should be avoided as overall DNA yields were reduced as well as the detection of bacterial taxa. Overall, we provide solutions for processing and storing human stool samples that reduce variability of microbiome data. We recommend that stool is frozen within 15 minutes of being defecated, stored in a domestic frost-free freezer for less than three days, and homogenized prior to DNA extraction.

## Results

### Subsampling of stool results in high variability of bacterial taxa detected via qPCR

**Fig 1:**
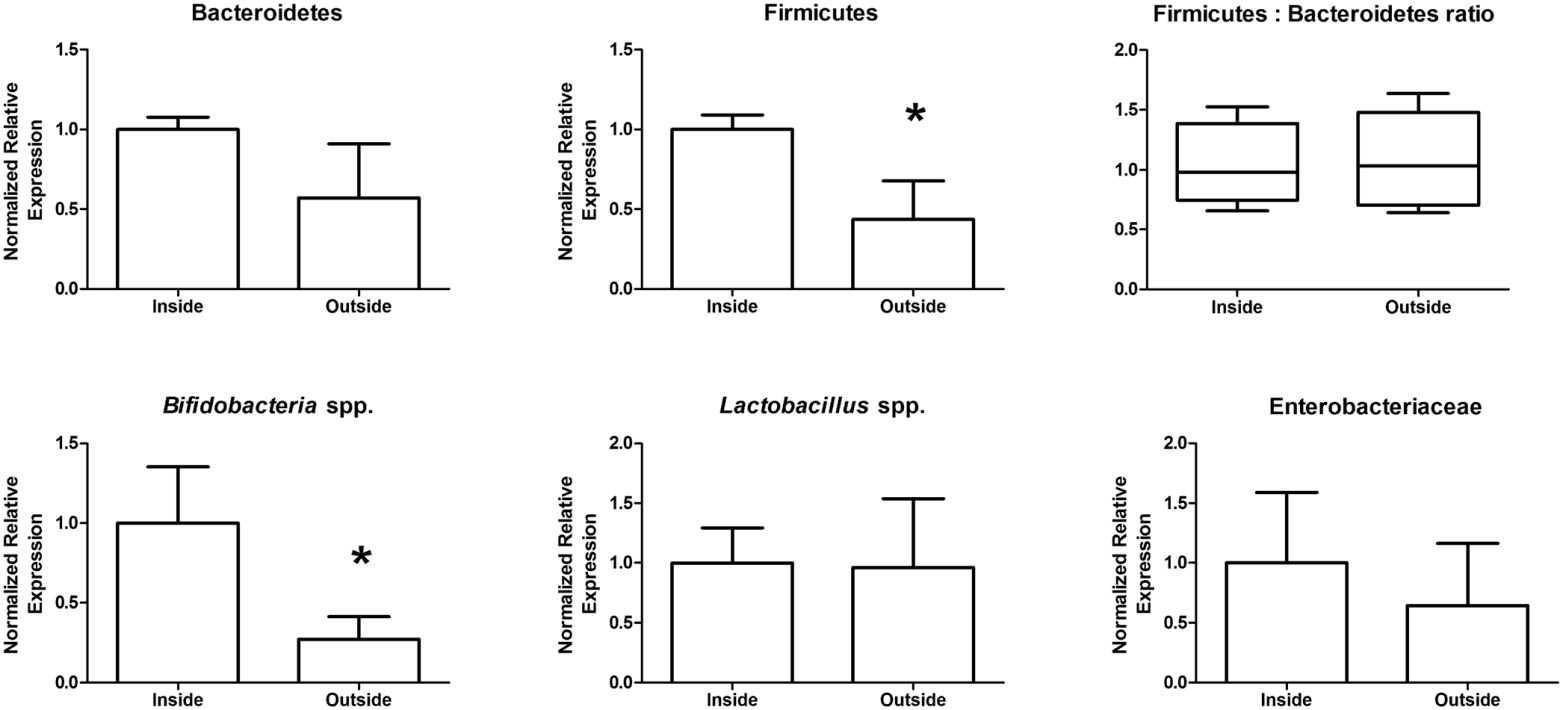
Bacterial taxa abundance differ in the inside compared to the outside microenvironments of stool. A single stool was subsampled five times from the inside environment and five times from the outer environment, DNA was extracted and used to compare bacterial taxa via qPCR. Firmicutes and *Bifidobacteria* spp. were decreased in the outside microenvironment compared to the inside microenvironment of the stool. *, p = 0.03.

The goal of our study was to generate reproducible qPCR detection of bacterial taxa present in a single stool sample. We compared subsamples of single stools from four healthy adults by examining prominent members of the gut microbiota via qPCR using specific primers to each bacterial taxa. We observed that subsampling of stool resulted in large variation in bacterial taxa abundance in each of the four subjects (Table 1; non-homogenized subsamples). While subject #4 demonstrated the largest variance in each of the taxa examined, subject #1–3 showed large variance in at least one of the taxa examined (Table 1; non-homogenized samples underlined). Overall, the mean of the variances for each taxa examined ranged from 393–2.3E13 (Table 1; non-homogenized samples in bold). Considering the oxygen tension would affect the growth of facultative and strict anaerobic bacteria differently, we hypothesized that the source of variation could be due to various microenvironments harbouring different bacterial populations in stool. To test this hypothesis, we compared taxa from subsamples taken from either the outer or inner microenvironments. Indeed, inner and outer regions of the stool generated significantly different results in the case of Firmicutes and *Bifidobacterium* spp. (Fig 1). Overall, these results suggest that subsampling of stool results in large variability of gut microbiome data due to different microenvironments harbouring various taxa within an individual stool.

**Table 1:** Homogenized stool subsamples have less variance compared to non-homogenized stool subsamples. Mean, standard deviation and variance values for each of the bacterial taxa detected via qPCR collected from four different subjects`stool subsamples that had been homogenized on liquid nitrogen or not homogenized. Levene's p-values are reported where a significant p-value denotes whether variance is significantly different between groups; the p value is italicized if the non-homogenized subsamples (underlined) have significantly higher variance compared to the homogenized subsamples. The averaged mean, standard deviation and variance between the four subjects is included in the right column (bolded text; large variance seen in non-homogenized subsamples).

		Subject 1			Subject 2			Subject 3			Subject 4		Mean of Variances	
	Homo	Non-Homo	Levene's	Homo	Non-Homo	Levene's	Homo	Non-Homo	Levene's	Homo	Non-Homo	Levene's		Not
			p-value			p-value		Homo	p-value			p-value	Homo	Homo
Bacteroidetes														
mean	1.0000	2.2386		1.0000	53.7730		1.0000	0.7740		1.0000	17.9636		0.1725	2838.7843
standard dev.	0.4106	1.6471		0.2850	106.5018		0.3172	0.7031		0.5828	3.0477		0.1175	5669.2395
variance	0.1686	2.7129	*0*.*00938*	0.0812	11342.6417	**<** *0*.*0001*	0.1006	0.4944	0.1152	0.3396	9.2883	**<** *0*.*0001*	0.0138	**3.2140E+07**
Firmicutes														
mean	1.0000	0.5534		1.0000	3.5658		1.0000	1.6151		1.0000	21.6180		0.2516	16.9679
standard dev.	0.2930	0.1061		0.2393	6.8001		0.6916	1.5468		0.6205	4.3847		0.2117	21.3075
variance	0.0858	0.0113	0.03752	0.0573	46.2419	**<** *0*.*0001*	0.4783	2.3927	0.1103	0.3850	19.2258	**<** *0*.*0001*	0.0448	**454.0091**
*Lactobacillus* spp.														
mean	0.0000	0.0000		1.0000	3.4068		1.0000	8.9849E-04		1.0000	9.7726		2.0585	11.8892
standard dev.	0.0000	0.0000		1.7316	2.5019		2.2340	1.4346E-03		0.4948	6.4263		2.3810	19.8261
variance	0.0000	0.0000	n/a	2.9983	6.2597	0.5060	4.9907	2.0580E-06	**<** *0*.*0001*	0.2449	41.2971	**<** *0*.*0001*	5.6692	**393.0737**
*Bifidobacteria* spp.														
mean	1.0000	0.8844		1.0000	0.0050		1.0000	1.3687		1.0000	10.0061		0.6699	15.8881
standard dev.	1.3259	0.5309		0.6178	0.0069		0.1896	1.1055		0.7099	7.8771		0.7520	30.7779
variance	1.7580	0.2819	0.04126	0.3816	0.0000	**<**0.0001	0.0359	1.2221	*0*.*00567*	0.5040	62.0483	*0*.*0136*	0.5655	**947.2795**
Enterobacteriacaea														
mean	1.0000	6.1151		1.0000	1587.8034		1.0000	9.7757		1.0000	11.0038		1.4519	2401532.9691
standard dev.	0.6412	13.6737		2.2361	3099.2814		0.1743	13.7178		0.6050	14.5375		2.3715	4802674.9470
variance	0.4111	186.9692	*0*.*00001*	5.0000	9605545.3895	**<** *0*.*0001*	0.0304	188.1773	**<** *0*.*0001*	0.3661	211.3403	**<** *0*.*0001*	5.6240	**2.3066E+13**

### Homogenization of stool reduces variability of bacterial taxa detected via qPCR

**Fig 2:**
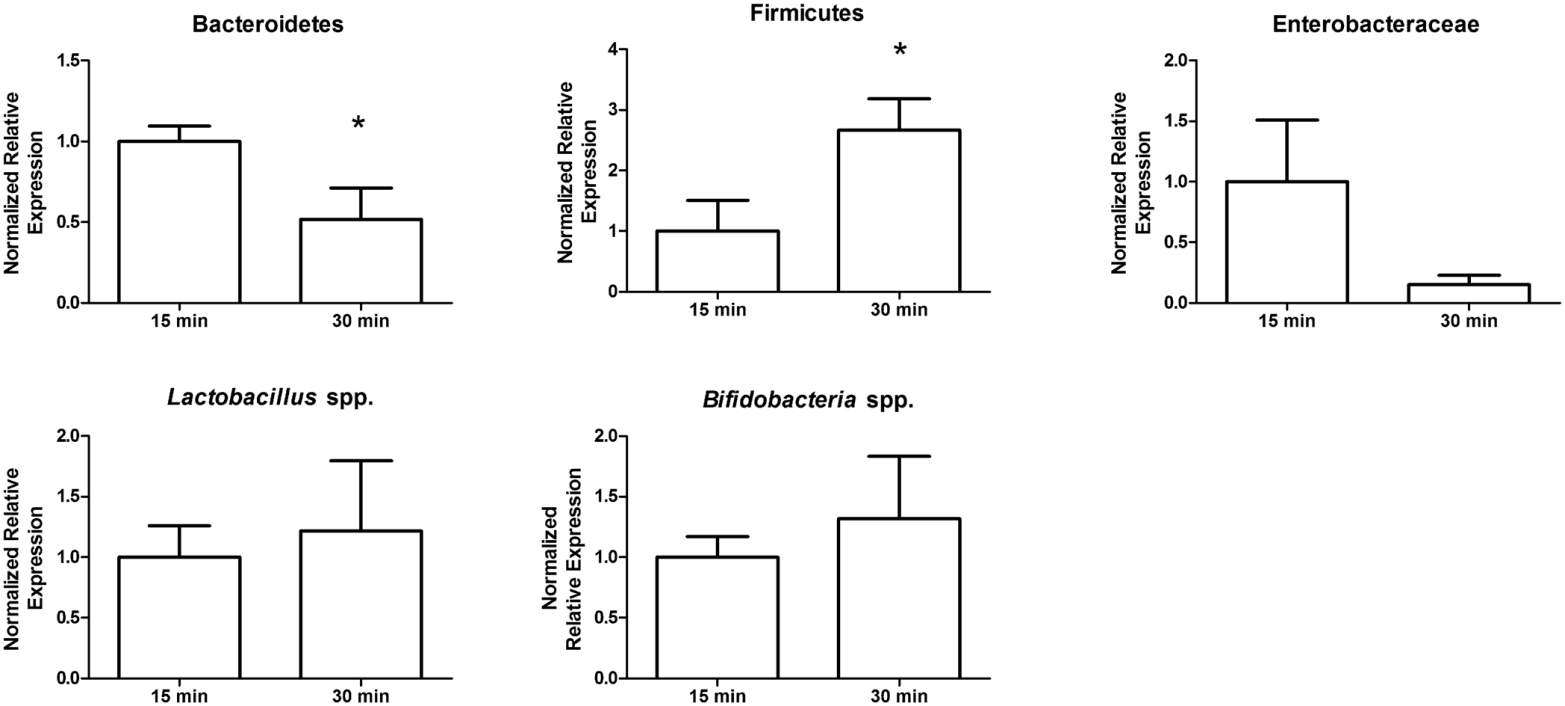
The mean variances of bacterial taxa are lower in homogenized subsamples compared to non-homogenized stool subsamples. The variance values were calculated for each of the bacterial taxa tested using qPCR from five subsamples where the stool was homogenized by crushing on liquid nitrogen into a fine powder and compared to stool not homogenized. The mean variance was calculated by taking the average of the variances determined for each bacteria taxa from the four subjects that were examined.

To determine if homogenizing stool samples would reduce the large variability found in subsampling stool, we ground frozen (-80°C) whole stool samples in liquid nitrogen using a mortar and pestle until the sample was a fine powder. We subsampled the frozen powder and extracted DNA for downstream qPCR detection of bacterial taxa. We found this technique significantly reduced the variance of bacterial taxa in all subjects (Table 1; italicized p-values denote significantly reduced variance in homogenized samples compared to non-homogenized samples). Overall, the mean of the variance for each taxa examined was reduced in the homogenized subsamples when compared to the non-homogenized subsamples (Fig 2). These results suggest that the variability in gut microbiome data can be improved by homogenizing the stool prior to subsampling.

### Storage of stool at room temperature affects qPCR detection of bacterial taxa

**Fig 3:**
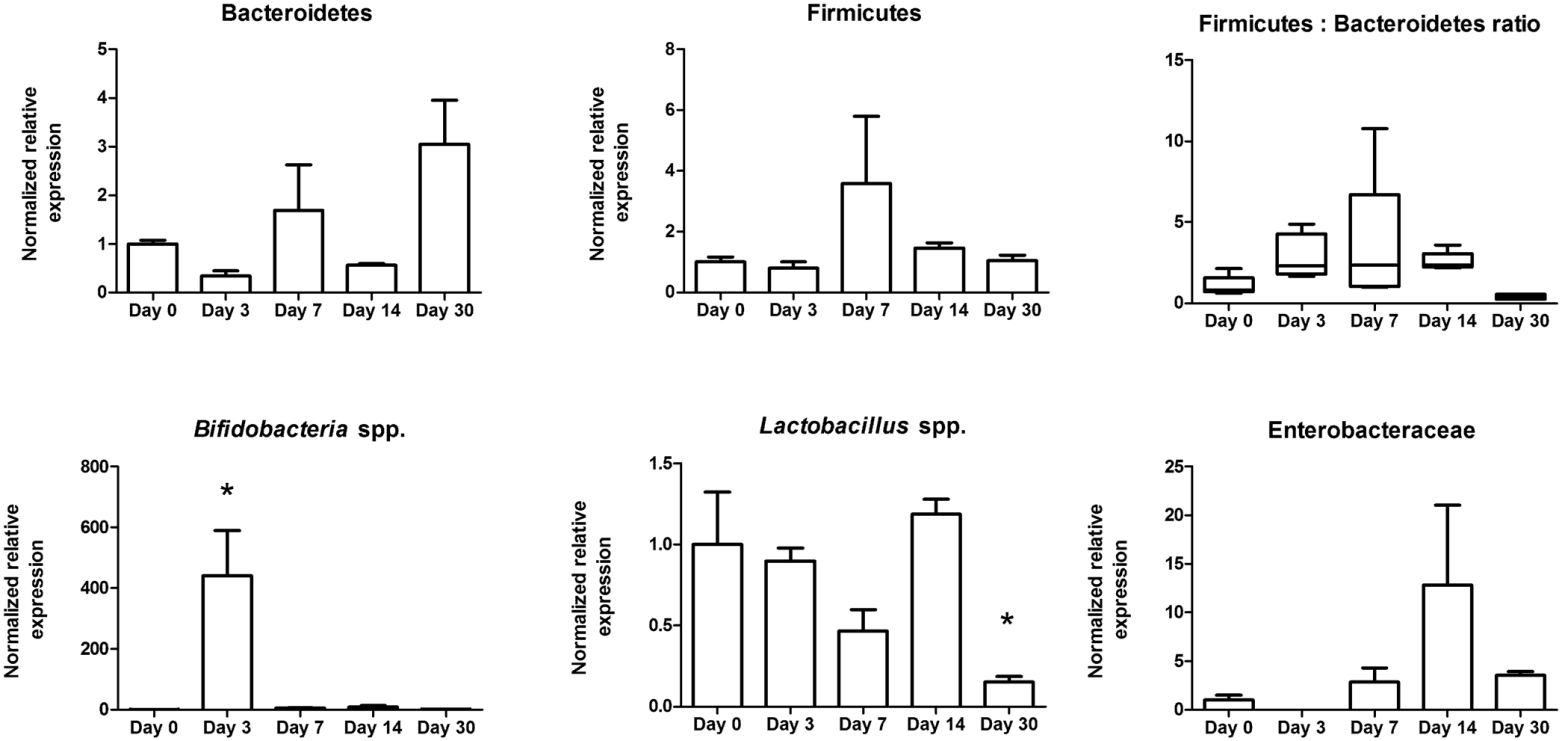
Stool storage at room temperature alters the abundance of bacterial taxa. Ten subsamples from the same stool were either stored at room temperature for 15 minutes or for 30 minutes, followed by DNA extraction and used to compare bacterial taxa via qPCR. Bacteroidetes detection decreased after 30 minutes at room temperature, whereas Firmicutes increased after 30 minutes. *, p > 0.05.

Sample storage at room temperature for a short period of time is important to sample collection procedures since it gives participants the opportunity to produce a sample privately. To determine how long stool may be left at room temperature prior to freezing without compromising the integrity of downstream DNA detection, we analysed stool samples which had been left at room temperature for 15 or 30 minutes. We found significant differences in the major phyla of the gut, Bacteroidetes and Firmicutes, after 30 minutes compared to 15 minutes of storage at room temperature (Fig 3). These results suggest that the stool samples should be frozen within 15 minutes post-defecation.

### Storage of stool in a domestic frost-free freezer affects qPCR detection of bacterial taxa

**Fig 4:**
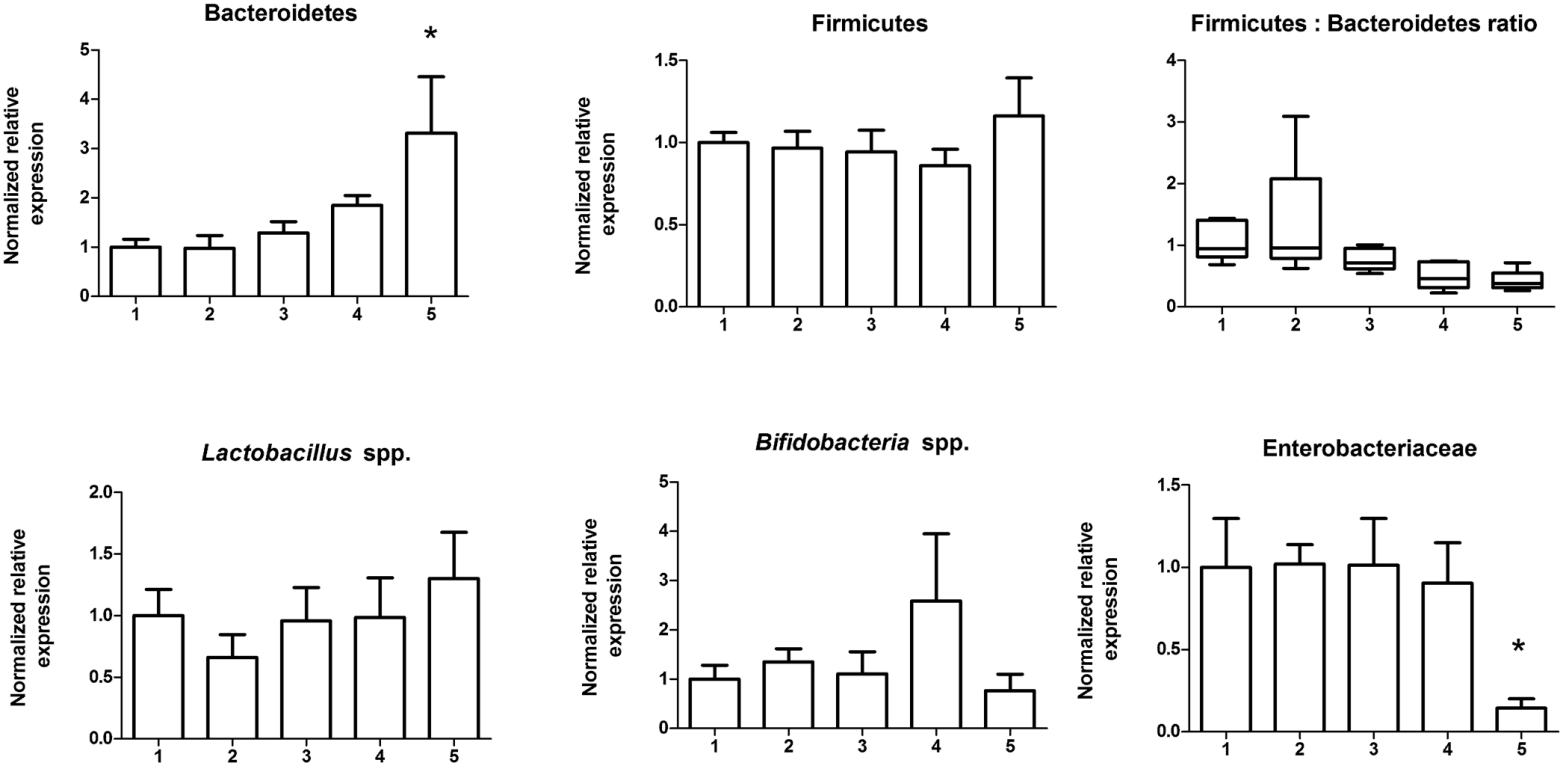
Stool storage in a domestic frost-free freezer affects the abundance of bacterial taxa. A homogenized stool sample was stored in a domestic freezer for 0, 3,7,14, and 30 days, DNA was extracted and used for qPCR to compare bacterial taxa abundance. All bacterial taxa tested showed some change in abundance by day 30. *, p < 0.05.

Sample storage in a domestic freezer is a practical solution for storing faecal samples prior to pick-up from participants. However, it is unknown how a domestic freezer, which typically ranges in temperatures from -20°C down to -2 in a 24 hr frost free cycle, affects the accuracy of downstream DNA-based bacterial taxa detection. To determine an appropriate temporary storage time, a single stool sample was homogenized and stored in a domestic frost-free freezer at for up to 30 days (Fig 4). We used day 0, or the just defecated sample, as the control and set this arbitrarily at 1 to compare the relative expression of a particular bacteria in stool that had been stored for either 3, 7, 14 or 30 days in the freezer. All bacterial taxa examined demonstrated a shift in relative expression compared to day 0 after storage in a domestic frost-free freezer. Bacteroidetes decreased significantly by day 14 while Firmicutes decreased by day 3 of storage although only at day 7 was the Firmicutes:Bacteroidetes ratio altered. Both *Bifidobacteria* spp. and *Lactobacillus* spp. decreased significantly by day 30 and Enterobacteriaceae detection increased significantly by day 14. Overall, these results suggest that a stool sample should be stored in a domestic frost-free freezer for less than 3 days.

### Freeze-thaw has little effect on qPCR detection of bacterial taxa

**Fig 5:**
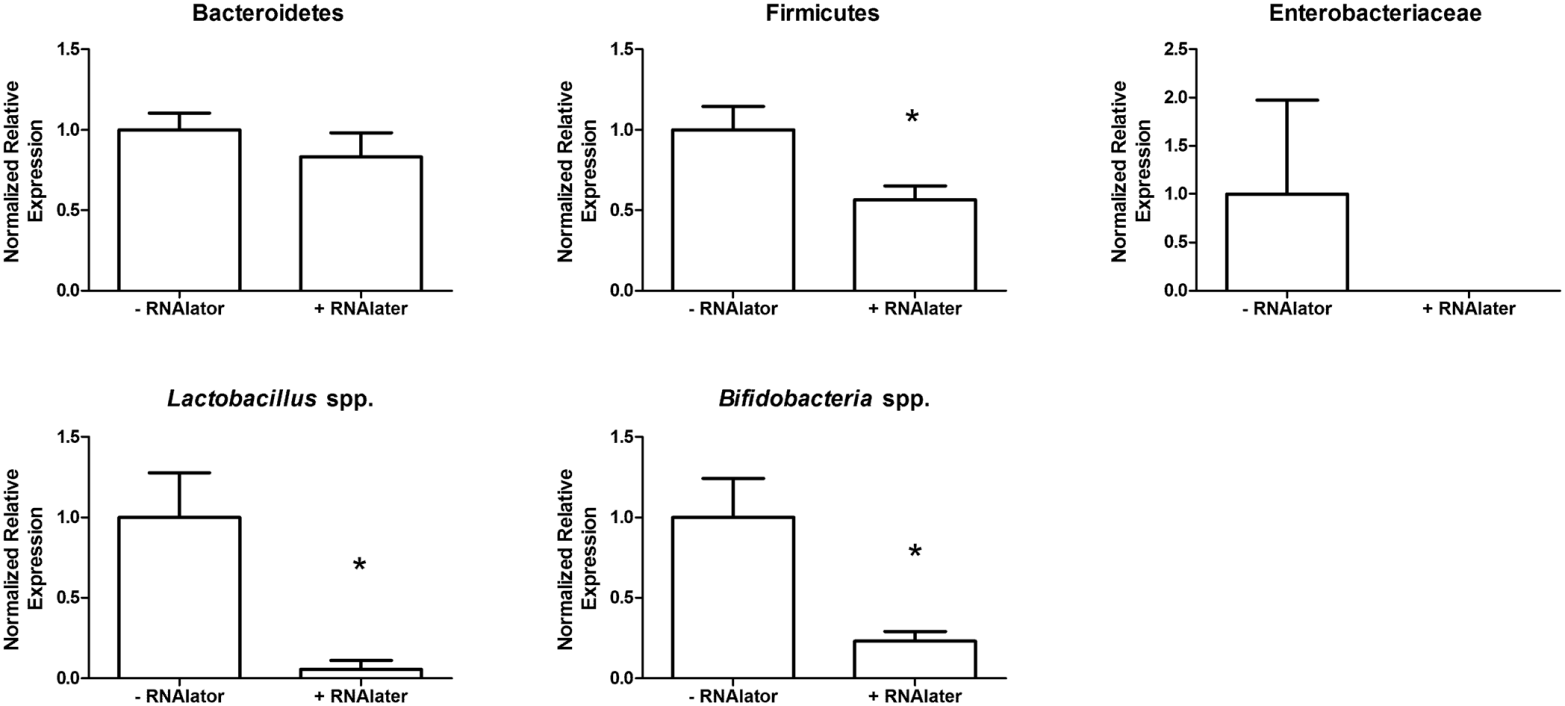
Freeze-thawing stool up to four times does not affect bacterial taxa abundance. A homogenized stool sample was subject to a series of up to five consecutive full freeze-thaw cycles, DNA was extracted and used for qPCR to compare bacteria taxa abundance. There were no changes of bacterial taxa abundance until the 5^th^ freeze thaw cycle where Bacteroidetes were increased and Enterobactericeae decreased. *, p < 0.05.

To determine the effects of thawing and re-freezing a sample we tested bacterial taxa abundance after up to four consecutive complete freeze-thaw cycles. Samples were first homogenized to ensure that the freeze-thaw treatment was generating the variation detected rather than the inherent variation in an un-homogenized stool sample. Each subsample was thawed for seven minutes and then snap frozen in liquid nitrogen per cycle. We found significant changes to detection only after four cycles of freeze-thaw where Enterobacteriaceae decreased and Bacteroidetes detection increased (Fig 5). Notably, we found significantly more variation in data when thawing cycles were beyond ten minutes (data not shown). Overall, these results suggest that a stool sample can be freeze-thawed up to four times.

### RNAlater does not preserve microbial DNA in stool

**Fig 6:**
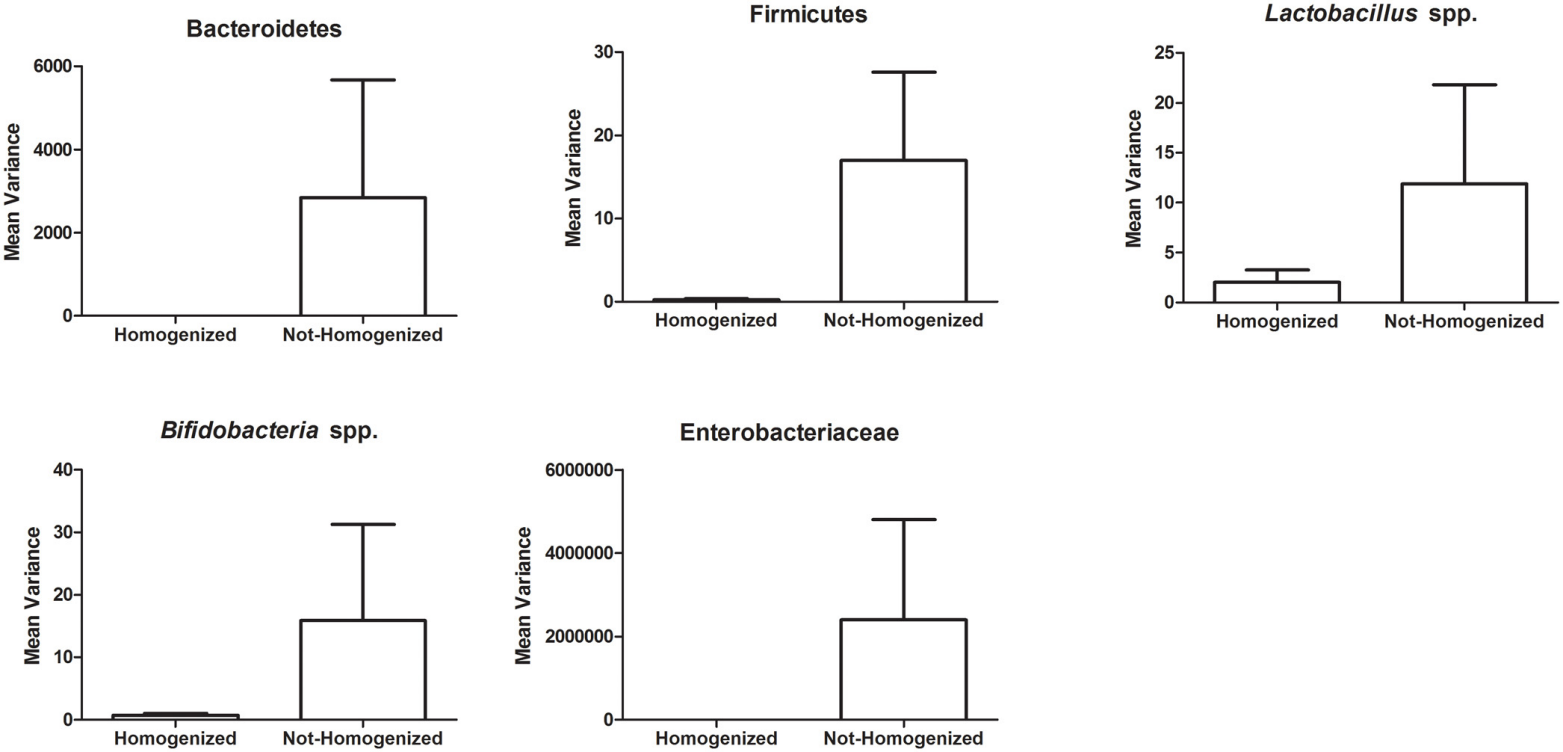
Stool stored in nucleic acid stabilizer prior to processing did not protect against bacterial taxa changes. Stool was either stored with or without RNAlater (Qiagen) prior to freezing and then processing stool samples. Detection of Firmicutes, *Lactobacillus* spp. and *Bifidobacteria* spp. was reduced after storage in RNAlater. *, p = 0.05.

To determine if we could improve the stability of the microbial nucleic acids in stool we stored stool with and without RNAlater. We found that RNAlater reduced the yield of DNA from stool samples (Table 2). As well, RNAlater resulted in reduced the abundance of all bacterial taxa examined (Fig 6). Overall these results suggest that RNAlater does not improve storage of stool samples.

**Table 2:** RNAlater reduces DNA yields from stool samples. DNA extracted from samples not stored in RNAlater was significantly greater (p<0.0001) than samples stored in RNAlater.

	RNAlater	no RNAlater
	55.10	83.70
DNA	18.30	252.50
concentrations	5.90	260.50
	85.40	266.30
	36.00	213.20
	28.50	234.30
mean	38.20	218.42
standard deviation	28.47	68.78

## Discussion

The overarching goal of human gut microbiome research is to represent the human gut environment, but current practice may be falling short of this goal without the consideration of stool processing and storage conditions. Indeed, high variability within stool has been identified as a challenge in data interpretation in the field [4,7]. The purpose of this study was to generate recommendations for the handling, storage and processing of faecal samples for intestinal microbiome analysis in human health research. By far the most important recommendation generated by this study is to homogenize the entire faecal sample prior to analysis. Furthermore, based on the data presented here, we recommend that human stool samples used for DNA-based microbial community analysis are frozen within 15 minutes of being defecated, and stored in a domestic frost-free freezer for less than 3 days. We suggest that sample handling of human stool should be standardized to reduce the large variability seen in microbiome analyses and to facilitate comparisons or pooled analyses of results from various studies.

In this study, we identified a reliable homogenization method for reducing variability and capturing the abundance of bacterial taxa in a single stool sample by grinding an entire frozen stool sample in liquid nitrogen prior to subsampling for DNA extraction. Homogenization creates a faecal powder, which can be stored in aliquots at -80°C for downstream analysis. This simple homogenization step will ensure that samples generate consistent microbial community composition reflective of the entire stool and not the microenvironment from which was subsampled. Considering, a microbial signal is a small difference in amounts of bacteria detected [11], the method of preparation of stool is relevant in interpretation of large data sets like those generated by the Human Microbiome Project (HMP) [12]. The current HMP protocol is to aseptically remove a subsample of stool directly into a lysis buffer for DNA extraction [12]. Since we found that subsampling the stool results in large microbial variation and that the inner and outer microenvironments of stool contain different microbes in a single stool, we believe subsampling of stool is unlikely to represent the gut microbiome ecosystem and may result in the inability to draw accurate conclusions from data. Indeed, only a few studies have reported homogenizing faecal samples prior to DNA extraction [13–15]. In support of our findings presented here, insufficient homogenization has been concluded to be a bias in metagenomics analysis [16]. Large variation of gut microbes have been evident even within the same individual at different times [17,18]. Overall, we suggest this variability is most likely due to subsampling stool and the lack of homogenization prior to DNA extraction.

The variation seen in human gut microbiome data could have major consequences to the interpretation and thus, understanding of the role of the gut microbiome in disease. For example, Finucane *et al* used HMP data to conclude that there is no microbial taxonomic signature associated with obesity [19], yet this is in conflict with numerous scientific evidence in humans and rodent models that support that microbes influence obesity [11,20–26]. While many of these studies are correlative, faecal transplantation demonstrates that obesity is transmissible in both humans and rodents which lends credence to the importance of the microbiota in the obesity phenotype [11,27]. Of importance, many of the above obesity studies had pointed out the altered ratio of Firmicutes to Bacteroidetes where obese children have been shown to have an increased ratio [24]. Considering our results reveal large variation of gut microbiome data is associated with subsampling as well as the effect of storage on stool sample microbes, it is critical that human stool processing be standardized.

Another important consideration in gut microbiota analysis of human stool is storage, and in particular in a domestic frost-free freezer since participants can easily store their sample at home until the researchers can arrange for sample pick up. Our results reveal impacts on bacterial taxa abundance where mostly decreasing relative expression of bacteria is found after storage with sample storage in a domestic freezer beyond three days. One exception to this trend was for Enterobacteriaceae, which appeared to increase and although unknown, this may reflect changes in oxygen tension may be one explanation. Alternatively, this could be a reflective of relative abundance qPCR which normalizes the specific taxa being looked at to the total bacteria. As an alternative to storage in a freezer, collected stool can be immediately stored in ethanol prior to silica desiccation [28,29].

While there are conflicting studies with regards to room temperature storage of human stool [8,13], Nsubuga *et al* (2004) found a negative correlation between the amount of DNA extracted from mountain gorilla faecal samples and the maximum temperature at the time of sample collection leading to their conclusion that faecal samples should be immediately frozen to preserve DNA [29]. Similarly, we find that room temperature storage for up to 30 minutes results in bacterial taxa alterations highlighting the importance of freezing stool immediately post-defecation.

Other concerns in the handling of faecal samples include the impact of freeze-thaw cycles on microbial detection. Studies have warned against freeze-thawing stool samples [16,30], however, we found that freeze-thaw can be employed for up to four cycles before bacterial taxa appear to change. Finally, while a nucleic acid stabilization agent like RNAlater has been previously used to store stool samples [29], there have been no studies that directly compare RNAlater treated samples with directly frozen samples for DNA yield and taxa comparisons. We found that samples treated with RNAlater and compared to frozen untreated samples did not preserve the DNA during sample preparation, processing and storage and instead, we found this reagent degraded the DNA. Thus, we do not recommend using a nucleic acid stabilizer like RNAlater.

Human stool samples appear to produce variability leading to different results from study to study. We recommend stool samples are collected by 15 minutes post-defecation and can be stored up to 3 days in the participant’s domestic frost-free freezer followed by homogenization under liquid nitrogen prior to DNA extraction. Homogenized samples can undergo four freeze-thaw cycles. Adoption of these simple protocols could have a significant impact on the quality of human gut microbiome data.

## Materials and Methods

### Ethics

Faecal samples were collected from four healthy participants that were between the age range 20–40 years old (two female and two male subjects). No individuals sampled in this study had a medical history of antibiotic usage for over a year or illness six months prior. All procedures involving human subjects were approved by the University of British Columbia Research Ethics Board. Participants provided written consent, which is securely stored on UBC servers (whereas hard copies are stored in locked cabinets in secure lab space), as per UBC research policy.

### Stool preparation for non-homogenized and homogenized subsamples

Faecal samples were collected within 15 minutes of defecation and then frozen at -80°C in sterile 50 mL polypropylene conical tubes. For non-homogenized subsampling, a sterile spatula was used to put 180–220 mg of frozen stool chunks cut from the interior portion of an individual stool log and put into 2 mL polypropylene tubes. For homogenized subsampling, an entire frozen stool was manually ground to a fine powder in liquid nitrogen using a sterile mortar and pestle. A sterile spatula was used to put 180–220 mg of frozen powder into 2 mL polypropylene tubes. The samples were put into freezer storage boxes and stored at -80°C prior to DNA extraction. Samples were not thawed until extraction.

### Inside-outside subsampling

A single stool from subject # four was subsampled when fresh, and each sample frozen at -80°C separately. Five 180–220 mg subsamples were taken from the surface of the stool, and five subsamples were taken from within the stool, taking care to reduce exposure to ambient air as much as possible. The samples were snap frozen and stored at -80°C until DNA extraction. Samples were not thawed until extraction.

### Room temperature storage

A stool from subject # four was collected in sterile 50 mL polypropylene conical tubes and then stirred with a sterile spatula in the absence of liquid nitrogen immediately post-defecation. Starting from post-defecation, five subsamples were exposed to the remainder of 15 or 30 minutes of room temperature incubation and then snap frozen and stored at -80°C until DNA extraction. Samples were not thawed until extraction.

### Domestic freezer storage

A stool from subject # four was collected in sterile 50 mL polypropylene conical tubes and then homogenized with a mortar and pestle on liquid nitrogen immediately post-defecation. The homogenized stool sample was subsampled five times for each of five time points (0, 3, 7, 14 and 30 days) and stored in a common household domestic frost-free freezer (Frigidaire). DNA extraction at day 0 was used as the control for all other time point comparisons, and was extracted immediately post-homogenization whereas the other samples were removed from the freezer and DNA extracted on day 3, 7, 14, and 30. Samples were not thawed until extraction.

### Freeze-thaw treatment

A stool from subject # four was collected in sterile 50 mL polypropylene conical tubes and then homogenized with a mortar and pestle on liquid nitrogen immediately post-defecation. The homogenized stool was subsampled five times for each freeze-thaw cycle. Subsamples were treated to an additional series of up to four consecutive freeze-thaw cycles. Each series of aliquots was thawed for seven minutes then dropped into liquid nitrogen for instant freeze. These steps were repeated four consecutive times to get the one to four cycles of freeze thaw.

### RNAlater storage

A stool from subject # four was collected in sterile 50 mL polypropylene conical tubes and then homogenized in the absence of liquid nitrogen immediately post-defecation. Starting from post-defecation, five subsamples were stored with and without RNAlater at -80°C. Samples were not thawed until extraction.

### Microbial Analysis

Five subsamples for each taxa from each stool were bead-beated with a 3 mm metal bead in a Retsch MixerMill MM 400 homogenizer at 30Hz for 2 X two minute cycles. DNA was extracted using the Qiagen Stool Mini Kit, according to the manufacturer’s instructions. QPCR was performed in duplicates in a volume of 10 μl with Sso Fast Eva Green Supermix (Bio-rad Laboratories) on the Biorad CFX 96 real time PCR detection system. QPCR was run on Biorad CFX 96 real time PCR detection system (Biorad Laboratories, Inc) on standardized concentrations of DNA (40 ng/ul). Primers specific to the 16S rRNA region of bacterial taxa were used (Table 3). All primers were synthesized by the Integrated DNA Technology (IDT), Canada. The annealing temperature of all bacterial primers was 60°C. QPCR was conducted with a lid temperature of 105°C and cycling conditions of denaturation at 98°C for 2 minutes, followed by 39 cycles of denaturation at 98°C for 5 seconds and annealing at 60°C for 31 seconds. Relative expression values for bacterial taxa were normalized to total bacteria present, amplified using a universal eubacteria primer.

**Table 3:** List of primers used in this study.

Group targeted	Primer Sequence 5' to 3'	Amplicon size	Reference
Bacteroidetes	F: CGATGGATAGGGGTTCTGAGAGGA	238	[31]
	R: GCTGGCACGGAGTTAGCCGA		
Firmicutes	F: GGAGYATGTGGTTTAATTCGAAGCA	126	[31]
	R: AGCTGACGACAACCATGCAC		
Enterobacteriaceae	F: CATTGACGTTACCCGCAGAAGAAGC	195	[32]
	R: CTCTACGAGACTCAAGCTTGC		
*Lactobacillus* spp.	F: AGCAGTAGGGAATCTTCCA	341	[33]
	R: CACCGCTACACATGGAG		[34]
*Bifidobacteria* spp.	F: CGCGTCYGGTGTGAAAG	244	[35]
	R: CCCCACATCCAGCATCCA		
Universal Eubacteria	F: ACTCCTACGGGAGGCAGCAGT	174–199	[36]
	R: GTATTACCGCGGCTGCTGGCAC		

### Data analysis and statistics

Gene expression studies were calculated using the Biorad CFX Manager Software Program Version 3.1.1217.0823. Relative abundance was calculated by setting a chosen control to 1 and then comparing the test samples to this control. All statistics were performed using GraphPad Prism Version 5.01. For Table 1, we arbitrarily set the homogenized stool sample at 1 to compare the relative expression of particular taxa in stool that was non-homogenized. The mean values, standard deviation and variance are reported. Levene's test was used to determine whether variance differed between groups where significance levels were p<0.05. For Figs 1–6, comparisons of means were done with ANOVA's when homogeneity of variance was true, and Kruskal-Wallis when variances were not homogeneous. For the inner and outer subsampling experiment we arbitrarily set the inner region at 1 to compare the outer relative expression of particular taxa. Comparisons of treatments was determined using unpaired t-tests for comparing two groups with Welch's corrections applied when variances were unequal. For room temperature storage, we arbitrarily set the 15 minute samples at 1 to compare the 30 minute samples relative expression of particular taxa. Comparison of treatments was determined with unpaired t-tests with Welch's correction for Enterobacteriaceae, and unpaired t-tests for the remaining bacterial groups (Bacteroidetes, Firmicutes, *Lactobacillus* spp., and *Bifidobacteria* spp.). For domestic freezer storage, we used day 0, or the just defecated sample, as the control and set this arbitrarily at 1 to compare the relative expression of bacterial taxa in stool that had been stored for either 3, 7, 14 or 30 days in the freezer. Kruskal-Wallis One-ANOVA with Dunn's multiple comparisons were performed for all bacterial groups tested; all bacterial groups had unequal variances in this experiment. For freeze-thaw treatments, we used a sample that had not been frozen and set this arbitrarily at 1 to compare all freeze-thaw subsample to. Kruskal-Wallis one-way ANOVA with Dunn's multiple comparisons for Bacteroidetes, *Lactobacillus* spp., *Bifidobacteria* spp. and Enterobacteriaceae because these groups had unequal variances; whereas the Firmicutes were compared using one-way ANOVA with Dunnett's multiple comparison test. For RNAlater treatment, the sample not treated with nucleic acid stabilizer was set arbitrarily to one to compare the subsamples, which had been treated with nucleic acid stabilizer and comparisons were performed with unpaired t-tests for all bacterial taxa.
