# Correction: Methods for Improving Human Gut Microbiome Data by Reducing Variability through Sample Processing and Storage of Stool

**DOI:** 10.1371/journal.pone.0139529

**Published:** 2015-09-25

**Authors:** The PLOS ONE Staff

The images for Figs 2–6 are incorrectly switched. The image that appears as Fig 2 should be Fig 3, the image that appears as Fig 3 should be Fig 4, the image that appears as Fig 4 should be Fig 5, the image that appears as Fig 5 should be Fig 6, and the image that appears as Fig 6 should be Fig 2. The publisher apologizes for these errors. Please view Figs 2–6 in the correct order below.

**Fig 2:**
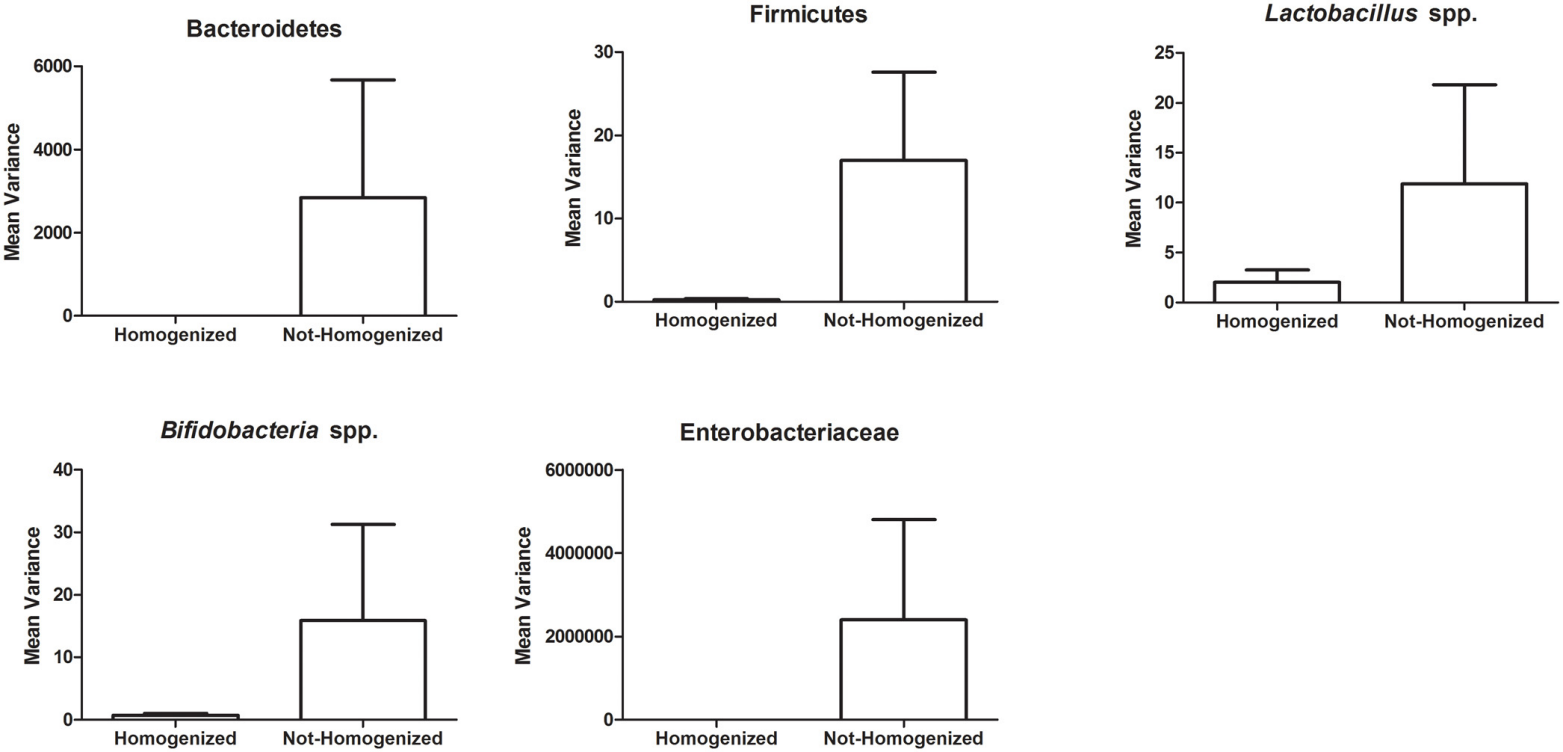
The mean variances of bacterial taxa are lower in homogenized subsamples compared to non-homogenized stool subsamples. The variance values were calculated for each of the bacterial taxa tested using qPCR from five subsamples where the stool was homogenized by crushing on liquid nitrogen into a fine powder and compared to stool not homogenized. The mean variance was calculated by taking the average of the variances determined for each bacteria taxa from the four subjects that were examined.

**Fig 3:**
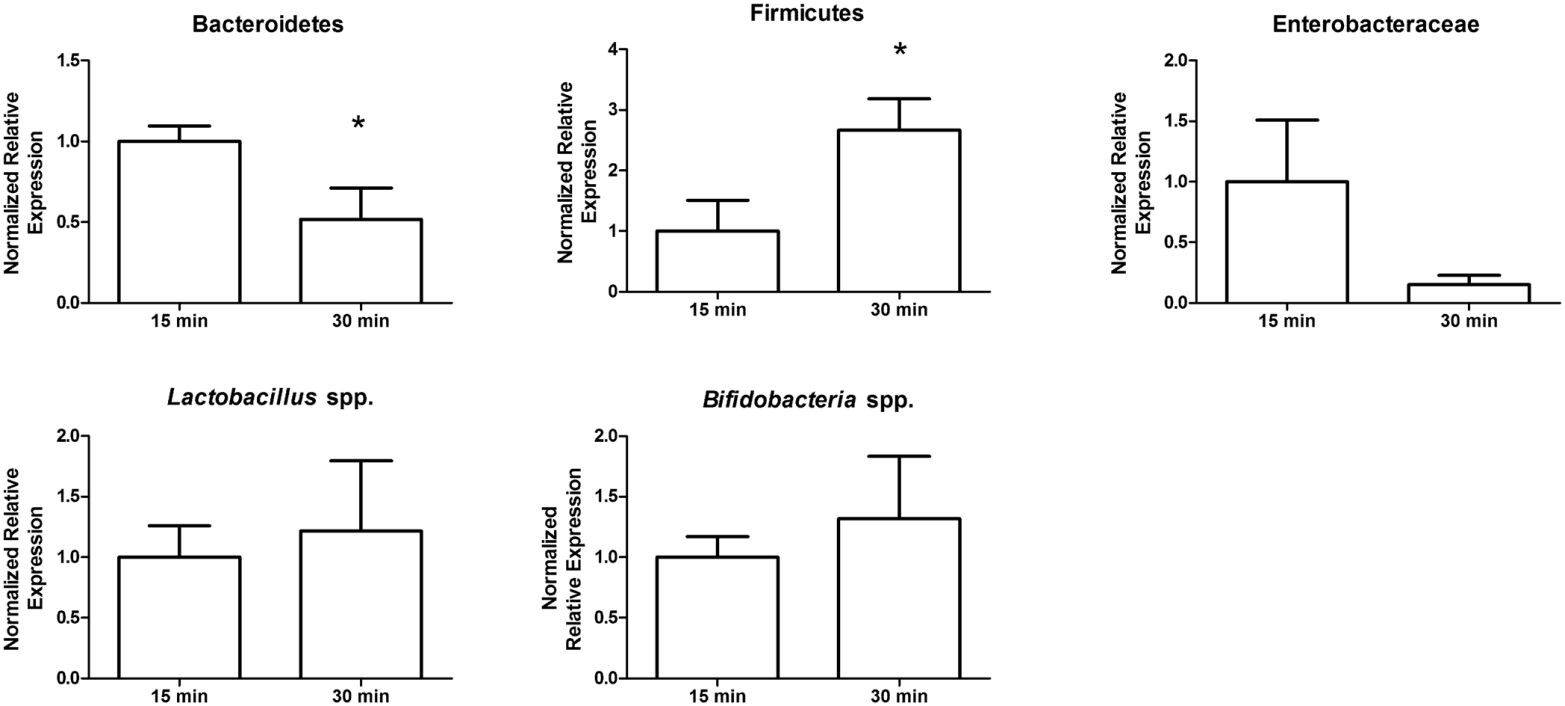
Stool storage at room temperature alters the abundance of bacterial taxa. Ten subsamples from the same stool were either stored at room temperature for 15 minutes or for 30 minutes, followed by DNA extraction and used to compare bacterial taxa via qPCR. Bacteroidetes detection decreased after 30 minutes at room temperature, whereas Firmicutes increased after 30 minutes. *, p > 0.05.

**Fig 4:**
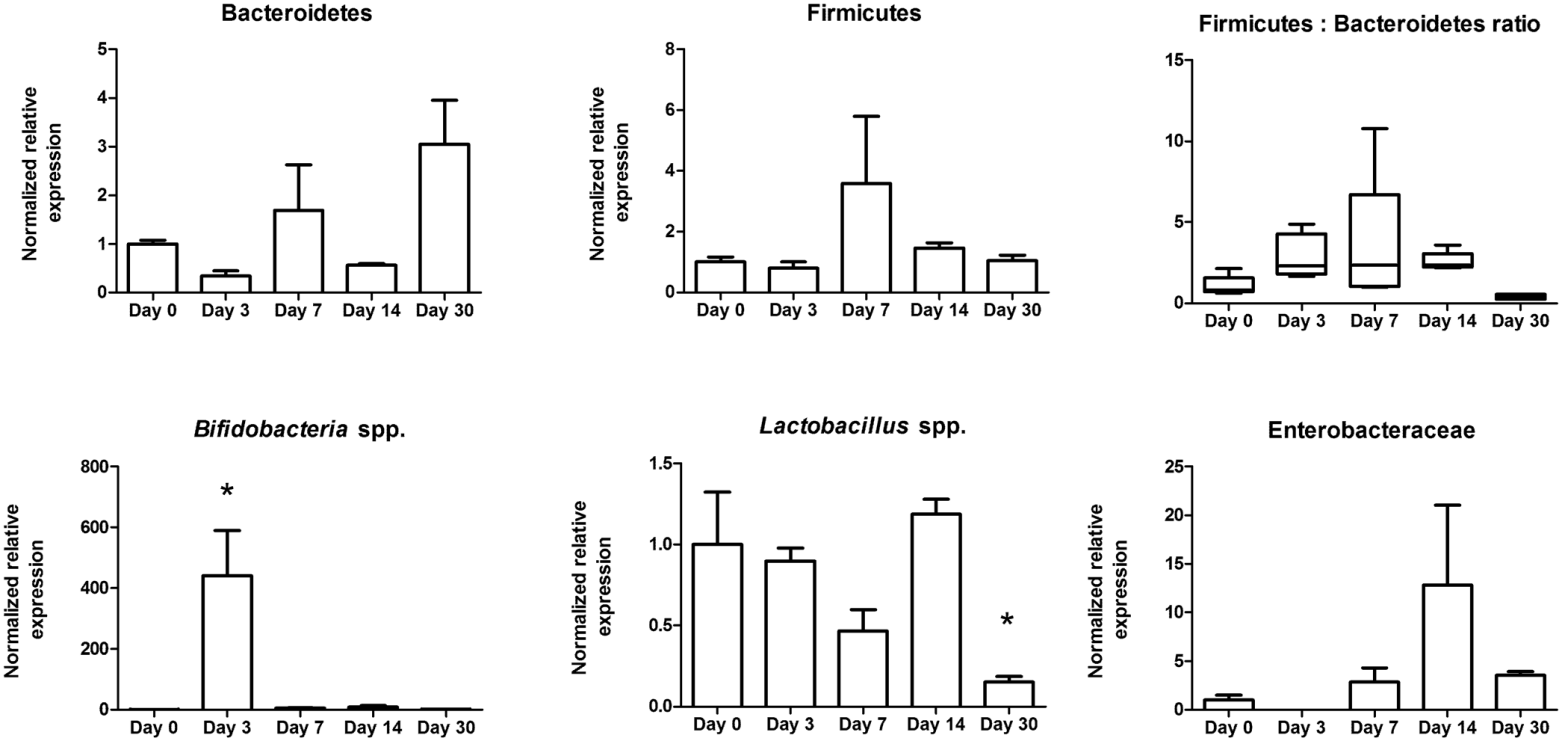
Stool storage in a domestic frost-free freezer affects the abundance of bacterial taxa. A homogenized stool sample was stored in a domestic freezer for 0, 3,7,14, and 30 days, DNA was extracted and used for qPCR to compare bacterial taxa abundance. All bacterial taxa tested showed some change in abundance by day 30. *, p < 0.05.

**Fig 5:**
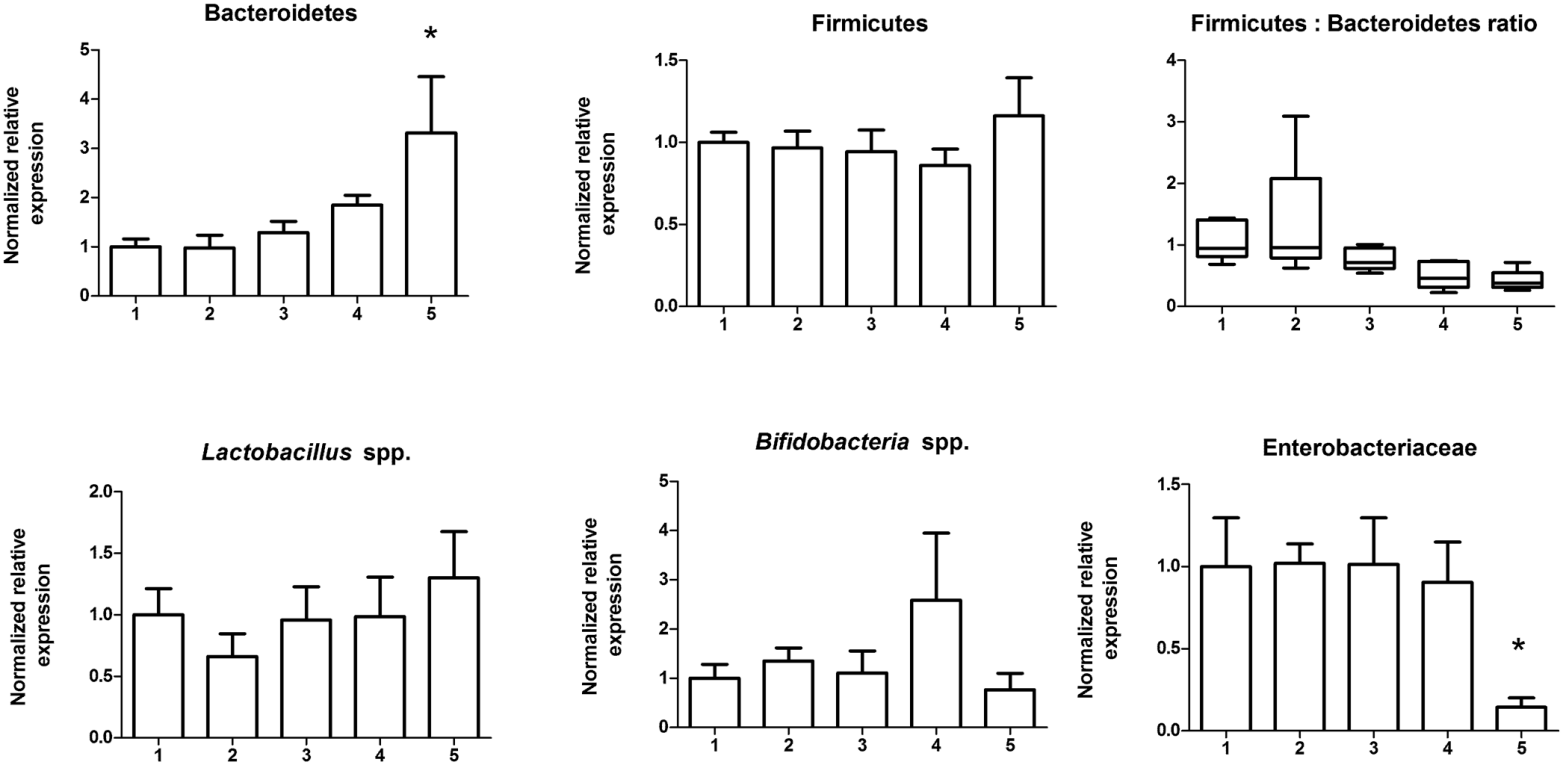
Freeze-thawing stool up to four times does not affect bacterial taxa abundance. A homogenized stool sample was subject to a series of up to five consecutive full freeze-thaw cycles, DNA was extracted and used for qPCR to compare bacteria taxa abundance. There were no changes of bacterial taxa abundance until the 5^th^ freeze thaw cycle where Bacteroidetes were increased and Enterobactericeae decreased. *, p < 0.05.

**Fig 6:**
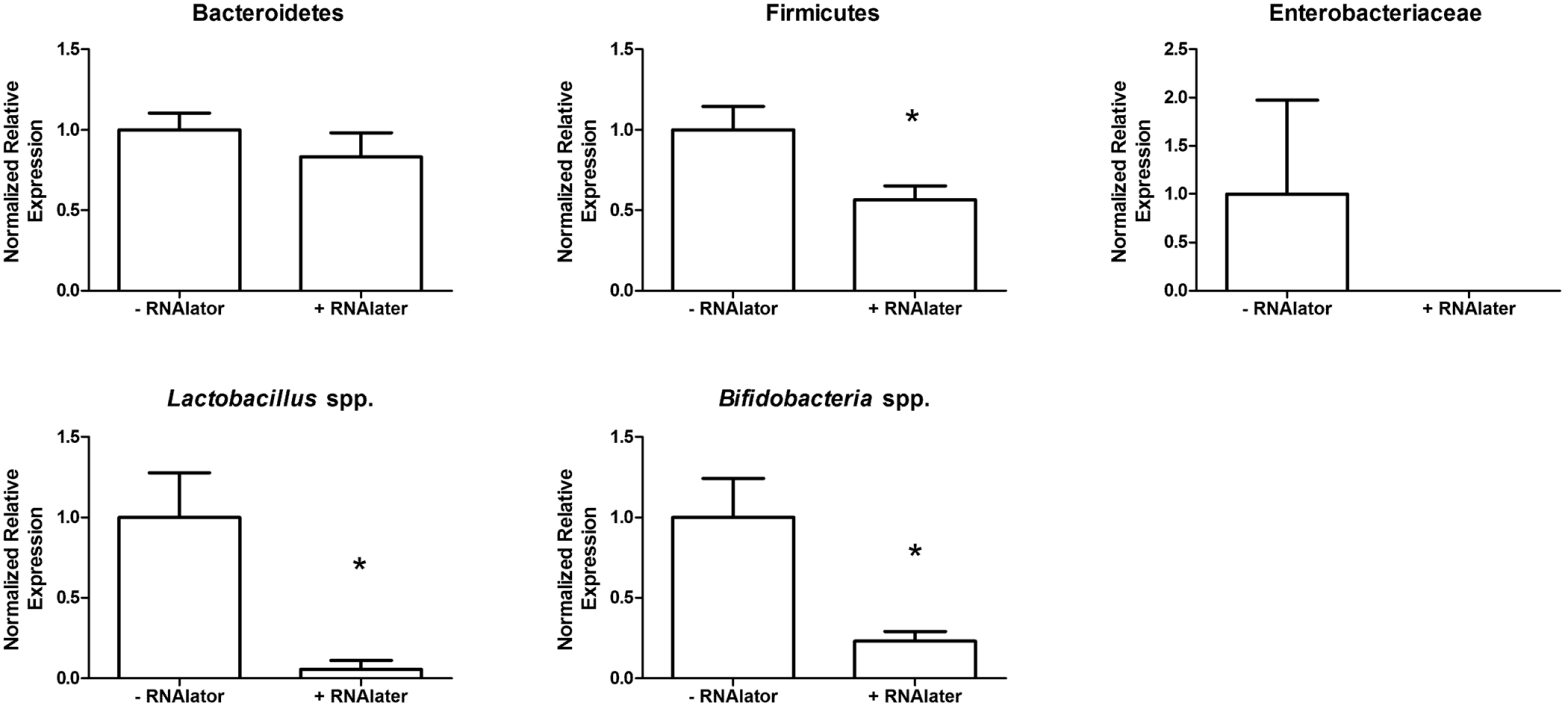
Stool stored in nucleic acid stabilizer prior to processing did not protect against bacterial taxa changes. Stool was either stored with or without RNAlater (Qiagen) prior to freezing and then processing stool samples. Detection of Firmicutes, *Lactobacillus* spp. and *Bifidobacteria* spp. was reduced after storage in RNAlater. *, p = 0.05.
